# Redox regulation of protein nanomechanics in health and disease: Lessons from titin

**DOI:** 10.1016/j.redox.2018.101074

**Published:** 2018-12-12

**Authors:** Elías Herrero-Galán, Inés Martínez-Martín, Jorge Alegre-Cebollada

**Affiliations:** Centro Nacional de Investigaciones Cardiovasculares (CNIC), Madrid, Spain

## Abstract

The nanomechanics of sarcomeric proteins is a key contributor to the mechanical output of muscle. Among them, titin emerges as a main target for the regulation of the stiffness of striated muscle. In the last years, single-molecule experiments by Atomic Force Microscopy (AFM) have demonstrated that redox posttranslational modifications are strong modulators of the mechanical function of titin. Here, we provide an overview of the recent development of the redox mechanobiology of titin, and suggest avenues of research to better understand how the stiffness of molecules, cells and tissues are modulated by redox signaling in health and disease.

## 1 The mechanobiology of muscle and titin

Muscle is the tissue where mechanobiological phenomena are most evident. Many of the cellular mechanisms of force generation and sensing were first described in muscle and then shown to work similarly in other tissues whose primary function is not mechanical [1]. The modulation and adaptation of muscle activity to different requirements is a matter of study since the beginnings of muscle physiology [2]. Only recently, a focus has been set on the nanomechanics of sarcomeric proteins as determining the mechanical properties of striated muscles and their regulation [3], [4], [5]. Sarcomeres are formed by highly ordered filaments to which regulatory and signaling proteins associate [6]. Active muscle contraction is achieved by sliding of arrays of myosin motors on actin-based filaments, whereas the giant protein titin is responsible for passive elasticity and stiffness [3].

**Fig. 1:**
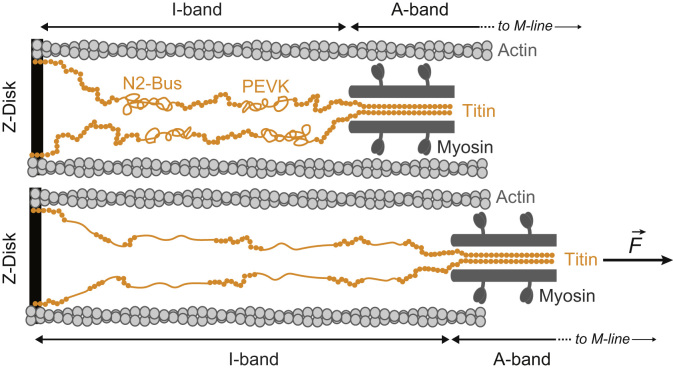
Schematic depiction of the contracted (top) and stretched (bottom) sarcomere (not to scale). Titin is colored in ocher, while other sarcomeric proteins appear in grey. Immunoglobulin-like domains are represented as filled circles, and the approximate positions of the unstructured N2-Bus and PEVK domains are indicated. Domains unfolded under force during stretching appear as extended lines in the bottom graph. The whole extension of the mechanically active I-band and the beginning of the A-band are delimited by arrows.

Titin, the largest protein encoded by the human genome (over 30,000 amino acids), is the paradigmatic example of an elastic protein that works under mechanical force [3]. It spans half the length of the sarcomere, from the Z-disk to the M-line, and ensures mechanical integrity of sarcomeres while providing stiffness to myocytes [7] (Fig. 1). Titin adjusts its total length at the elastic I-band to the needs of the functioning muscle. Two complementary mechanisms explain the elasticity of titin: entropic elasticity of unstructured regions (PEVK and the cardiac specific N2-B unique sequence, N2-Bus) and dynamic unfolding/refolding of its more than 100 constituent immunoglobulin (Ig) domains [5] (Fig. 1). When an Ig domain unfolds under force, around 100 amino acids are released, leading to increased contour length of the extended polypeptide. Hence, the protein domain becomes softer, whereas refolding has the opposite effect. The balance of folded vs unfolded domains is a key contributor to the overall elasticity and stiffness of titin and muscle tissue [5]. Although protein mechanical unfolding and folding reactions were first described for titin domains [8], [9], [10], now we know that these transitions are also involved in cellular mechanosensing and mechanotransduction in other proteins, such as talin and spectrin, to couple force-induced exposure of cryptic sites to downstream signaling [11], [12]. Here, we review recent work describing how redox biochemistry can modulate the mechanical properties of titin. We anticipate that pioneering work on titin will pave the way to the discovery of equivalent modes of mechanical regulation in other systems involved in mechanosensing, mechanotransduction and force generation.

The mRNA coding for titin is alternatively spliced, resulting in muscle-specific isoforms [3]. Isoforms differ in their total length and the ratio of folded domains to unstructured regions in the I-band, resulting in titin molecules with tailored elastic properties [3], [7]. However, we are just beginning to understand how changes in mechanical properties can be achieved in shorter timescales that are not compatible with the long protein turnover of titin [13]. A straightforward mechanism involves regulation of pre-existing titin molecules by posttranslational modifications (PTMs). Indeed, phosphorylation at the unstructured regions modulates the stiffness of titin [3], [7], and arginylation has been recently proposed to affect muscle stiffness, in part by altering the interactions of the A-band of titin with other proteins [14]. However, any modification that targets the mechanical properties of the more abundant, mechanically active Ig domains of the I-band can lead to more extensive changes in stiffness.

**Fig. 2:**
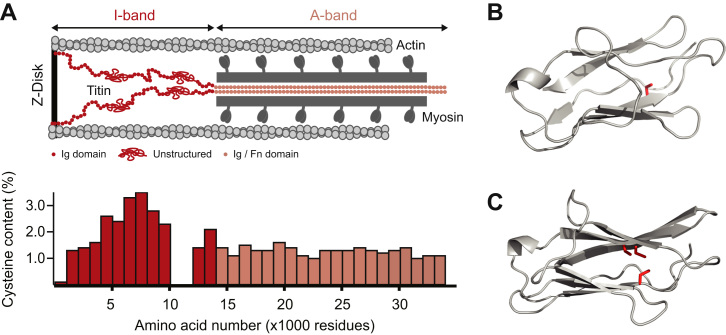
**A**: Representation of half a sarcomere (*top*, not to scale), indicating the positions of the I-band (red) and the A-band (beige) aligned with the cysteine content along the sequence of human titin (*bottom*) [15]. Immunoglobulin (Ig), unstructured and fibronectin III (Fn) domains are shown. **B** and **C**: 3-D homology models of immunoglobulin-like domains I15 (2620–2703) and I66 (8326–8414), respectively [15]. Cysteine side-chains are highlighted in red. Sequences correspond to uniprot entry Q8WZ42.

The I-band of titin contains a high proportion of cysteine residues, a major target of redox modifications in proteins [3], [15], [16], [17]. The majority of Ig domains in this region are above the 2.26% average cysteine content estimated for mammals [18] (Fig. 2A). Structurally, highly conserved cysteines in titin are buried in the fold of Ig domains, and can appear paired with other cysteine residues or not [15], [16] (Fig. 2B,C). The sulfur atoms of paired cysteines can engage in disulfide bonds, which are intramolecular covalent bonds that cannot be cleaved by physiological forces. As a result, disulfide-containing protein domains, as domains containing other covalent intramolecular bonds, cannot be completely extended by force during unfolding and therefore are stiffer than reduced counterparts [19], [20], [21], [22]. In the case of domains containing unpaired cysteines, mechanical unfolding can trigger their modification by reactive redox metabolites in the cytosol. Any such modification can introduce steric impediments to refolding, forcing the domain to remain in a soft, unfolded state [15]. Thus, redox PTMs of titin's cysteines appear as targets for the regulation of muscle stiffness.

## 2 Mechanical outcomes of redox PTMs

**Fig. 3:**
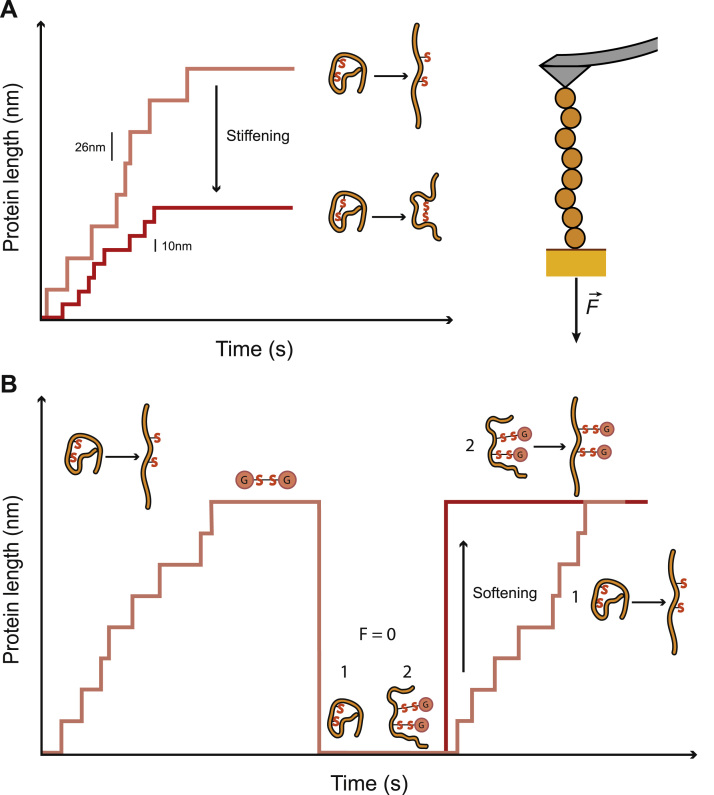
Schematic AFM traces showing the mechanical effects of redox PTMs. **A:** When a single polyprotein made of repetitions of one of titin's Ig domains is stretched under force in the AFM (*right*), 26 nm stepwise increases in protein length are detected, corresponding to individual domain unfolding events (*left*, beige line). If a disulfide bond is formed between two cysteines, the stiffening of the domains is manifested by their limited extension, showing 10 nm steps (red line) [16]. **B:** When a polyprotein is extended in presence of oxidized glutathione (G-S-S-G), cryptic cysteines become exposed to the reagent and might be modified. Intact domains are able to refold when force is relaxed, and their unfolding is detected again in a second probe pulse (1), whereas modified domains are unable to refold leading to immediate full extension of the polyprotein at high force (2) [15].

Oxidative and nitrosative signaling pathways have been described to modulate the mechanical properties of muscle, especially in the heart [23], [24], [25], [26]. Recent results suggest that the reverse may also be true, since redox signaling pathways can be triggered by physiological stretch [27]. When the nitroso-redox balance of cardiomyocytes is altered, such as during ischemia/reperfusion injury, changes in the mechanical properties of cardiac muscle occur [23], [28]. It has been reported that reversible redox PTMs of cysteine residues target key components of myocytes, such as the ryanodine receptor or protein kinase C [17], [29]. Some of these modifications have been observed in proteins with mechanical functions including titin, especially under situations of oxidative stress [24], [30], [31], [32]. However, the majority of these insights were obtained using bulk biochemistry and molecular biology techniques, which are useful to inspect biochemical regulation of proteins, but fall short at describing the function of elastic proteins that work under mechanical load. Force is a vector, defined not only by its magnitude but also by the point and direction of application. Since it is not possible to apply controlled forces to the billions of randomly oriented molecules in a test tube, classic bulk techniques cannot study the effect of mechanical forces on proteins. Only with the advent of single-molecule force-spectroscopy techniques has it become possible to examine how proteins behave under a pulling force [33]. In particular, force-spectroscopy by Atomic Force Microscopy (AFM) has proven the technique of choice to examine the mechanical effects on redox PTMs on protein domains [34]. In the AFM, a force signal is detected when a protein is tethered between a cantilever and a surface that can be retracted thanks to a piezo actuator. In single-molecule AFM experiments, fingerprinting is based on the use of engineered polyproteins made of repetitions of the domain of interest [34]. When pulled under a constant stretching force, polyproteins unfold in a step-wise manner, where every step in length marks the unfolding of one of the domains in the polyprotein (Fig. 3). Since all domains in the polyprotein are equal, all steps have the same size, enabling straightforward identification of recordings originating from single molecules. In AFM experiments, mechanical stability is probed by monitoring the force at which protein unfolding occurs. Refolding can be subsequently studied by quenching the force to low values and then pulling again at high forces to detect refolded domains [15].

### 2.1 Disulfide bonds limit protein mechanical extension

Perturbations that lead to formation of disulfides, such as incubation with H_2_O_2_, increase the stiffness of muscle [35], [36]. Conversely, the stiffness of isolated human heart myofibrils can be decreased by incubation with the reducing enzyme thioredoxin [35]. These results suggest that disulfide bonds can be established in titin. Indeed, the presence of disulfide bonds in the Ig domains of titin was observed for the first time in the crystal structure of a recombinant form of the I1 domain [37] and more recently in I69 [38]. Disulfide formation is easily captured by AFM pulling experiments. When a disulfide bond is formed, amino acids clamped between the residues forming the disulfide cannot contribute to the whole extension of the protein under force, resulting in protein stiffening that can be detected as shorter unfolding steps in the AFM (Fig. 3A) [22]. Pioneering AFM experiments showed that while disulfide formation in the N2-Bus region occurs readily under oxidizing conditions [35], oxidation of buried cysteines in recombinant *E. coli*-produced titin Ig domains is more challenging [39]. These results point to the existence of muscle-specific mechanisms of oxidation of titin domains.

Mechanically triggered disulfide isomerization has been described to occur in Ig domains containing a disulfide bond and a free cysteine [40]. When these domains unfold under force, the free cysteine can attack the disulfide, resulting in disulfide isomerization. Interestingly, sequence analyses show that 21% of titin's I-band Ig domains contain a triad of highly conserved cysteines that can engage in disulfide isomerization reactions (Fig. 2C) [16]. AFM experiments showed that oxidized triad-containing domains establish only one of the three potential disulfides, precisely the one that enables mechanically activated disulfide isomerization [16]. These experiments also showed that oxidized domains are less mechanically stable than reduced counterparts, but show higher refolding rates. Hence, when competing with reduced domains, oxidized domains are the first both to unfold and to refold. According to these results, it was proposed that oxidized domains in titin are primed for mechanical unfolding, enabling further extension of the polypeptide chain by disulfide isomerization reactions. Such multi-state extensibility of titin contributes to its versatile and adaptable mechanical properties [16]. Remarkably, computer simulations fed with kinetic data obtained with the AFM show that the magnitude of mechanical regulation by disulfide bonds in titin domains can be higher than the one achieved by phosphorylation of the random coil regions [16].

### 2.2 Redox PTMs introducing steric impediments to protein refolding

One of the end products of redox pathways is S-glutathionylation of cysteine residues [31], [41], [42], [43], which has been suggested to be responsible for altered sarcomeric function in infarcted murine hearts [30]. In this study, titin was identified as a target of S-glutathionylation, which may contribute to the functional alterations in the model. AFM experiments demonstrated that S-glutathionylation of cryptic cysteines induces softening of titin domains [15]. Mechanical unfolding of titin Ig domains in the presence of oxidized glutathione resulted in S-glutathionylation of buried cysteines by simple thiol-disulfide exchange [44]. S-glutathionylation led to inhibition of protein refolding and mechanical destabilization of parent domains (Fig. 3B). Both effects contribute towards reduced protein stiffness. Consistently, S-glutathionylation-induced softening of titin was accompanied by softening of skinned cardiomyocytes, in agreement with the widespread presence of buried cysteines in the Ig domains of titin [15] (Fig. 2). Although S-glutathionylation was the first S-thiolation modification whose mechanical effects were demonstrated, it is tempting to speculate that alternative redox modifications such as nitrosylation may induce similar effects via steric hindrance of refolding.

Interestingly, disulfide formation and S-thiolation of cysteines are interconnected, since S-thiolated cysteines can react with a second cysteine resulting in disulfide formation [44], [45], [46]. However, not all cysteine modifications are equally effective at inducing disulfide formation. Whereas S-glutathionylation does not favor disulfide bond formation [15], S-sulfenylation is a highly efficient mediator of disulfide formation provided side reactions are avoided [47]. Indeed, it has been shown that protein nanomechanics can be modulated by the specific nature of the S-thiolating agent [48], [49].

## 3 Implication of protein nanomechanics in organ homeostasis and disease

The mechanical properties of muscles must be tightly regulated to accommodate different physiological needs, such as during sleep, light physical activity or strenuous exercise. We are beginning to understand the molecular mechanisms behind this regulation. In this regard, grasping how sarcomeric proteins achieve their optimal elastic output is key to understand the regulation of the mechanical function of muscle.

Single-molecule AFM experiments have shown that redox PTMs induce dramatic changes to the mechanics of sarcomeric proteins. Disulfide bonds limit extensibility and lead to protein stiffening [16], [35], [40], whereas S-glutathionylation softens protein domains [15] (Fig. 3). These studies have provided for the first time a connection between redox biochemical reactions and mechanical function of protein domains. However, there is still room for technical improvement in the mechanical characterization of proteins. The range of physiological forces acting on proteins, in the order of tens of picoNewtons, is in the lower limit of AFM sensitivity. Alternative methods, such as Magnetic Tweezers (MT), achieve higher resolution at low forces, better stability, and can also operate in constant force mode [4]. The observation of the effect of disulfides in the mechanical properties of Ig domains at low forces has recently been achieved using MT [50].

Titin contains many domains with clustered cysteines, which most likely form disulfide bonds in vivo [16], [37]. However, the sarcomere is assumed to be a cytosolic structure whose highly reducing redox potential may not be compatible with disulfide formation [51], [52]. Although functional relevance can be deduced from strict conservation of cysteine residues and their role in the evolution of titin [15], [16], [53], studies addressing the existence of disulfides and other oxidative modifications of titin in vivo are needed. Identification of native redox PTMs in titin domains is a first step towards characterization of the mechanisms involved in their formation [46], [54], and would open the gate to explore the causes of its alteration in redox-related pathologies. Indeed, recent results demonstrate that titin-based stiffness of cardiomyocytes is increased in the early phases after myocardial infarction, but the underlying mechanisms are not fully understood [55]. In myocardial infarction, ischemia and reperfusion result both in stiffening of the myocardium and in oxidative stress that greatly alters redox signaling of myocytes [56], [57]. This stiffening is generally assumed to be caused by accumulation of extracellular matrix in the myocardium. However, a recently published study demonstrates that in the early phases after myocardial infarction, the intrinsic, extracellular-independent stiffness of cardiomyocytes is already increased, pointing to specific mechanical alteration of titin that can only be partially explained by phosphorylation changes [55]. We propose that variation of the redox state of titin may underlie maladaptive mechanical function of the myocytes after a myocardial infarction.

Mapping PTMs targeting native titin domains is necessary, but far from sufficient, to understand the complex modulation of titin's mechanical properties. Results so far show that two different kinds of oxidative modifications, i.e. disulfide formation and S-glutathionylation, lead to opposed mechanical effects (Fig. 3). It is very likely that both types of modifications target different titin domains at the same time. Hence, the overall effect of oxidative stress in the elasticity of titin is difficult to predict. Several factors add up to the complexity of titin's mechanical regulation by redox signals, such as mechanically activated redox signaling [27], crosstalk between disulfide formation and S-thiolation [48], position-dependent mechanical effects [58], the fact that redox reactions are themselves tuned by mechanical forces and depend on the folding status of parent domains [16], [46], [59], and that titin isoforms have different distribution of cysteines [16]. Despite the complexity of the system, it is possible to make informed predictions about the response of titin to environmental redox conditions in the context of a tissue by integrating data from single-molecule and muscle physiology experiments into free energy models and computer simulations [16], [19], [35], [50], [60]. It is thrilling to hypothesize that nature takes advantage of the complex redox regulation of titin to very precisely accommodate its mechanical properties to the physiological needs of the many muscles in the body, in a timely manner.

## 4 The emergence of redox mechanobiology

The demonstration that redox signals modulate the nanomechanics of titin opens the way to explore similar effects in other proteins that participate in mechanobiological processes. For example, myosin-binding protein C, a modulator of muscle contraction that works under mechanical load [61] can also establish disulfide bonds [62] and has been shown to undergo S-glutathionylation in mouse models of hypertension that cause diastolic dysfunction [31]. Cysteine residues that may modulate nanomechanics have already been identified in non-sarcomeric proteins with mechanical roles such as fibronectin [63], spectrin [12] and integrin [64]. We propose that redox control of protein nanomechanics is a widespread mechanism to control the output of mechanical circuitry in biology.

## Glossary

**Mechanosensing**: act by which a cell or tissue are able to perceive a mechanical stimulus.
**Mechanotransduction**: action by which a cell or tissue respond to a mechanical stimulus by transforming it into a biochemical signal.
**N2-B**: a cardiac-specific isoform of titin.
**Passive elasticity**: ability of a material to return to its original size and shape without energy consumption when an applied tension is released.
**PEVK**: a proline-aspartate-valine-lysine-rich unstructured region of titin.
**Polyprotein**: recombinant polypeptide made of repetitions of protein domains to be studied by single-molecule force-spectroscopy techniques.
**Softening**: decrease in stiffness.
**Stiffness**: resistance of a material to elongation or shortening by mechanical forces.
